# Revisiting Prinzmetal Legacy: The Structural Determinants of Coronary Spasm in Angina and Nonobstructive Coronary Arteries

**DOI:** 10.1016/j.jscai.2025.103862

**Published:** 2025-07-22

**Authors:** Rafael Gomes, Jorge Guimarães

**Affiliations:** Department of Cardiovascular Medicine, PROCAPE – Universidade de Pernambuco, Recife, Pernambuco, Brazil; Interventional Cardiology Lab, PROCAPE – Universidade de Pernambuco, Recife, Pernambuco, Brazil

We read with great interest the recent article by Bastiany et al,^1^ which offers important insights into the interplay between myocardial bridging (MB), moderate atherosclerosis, and epicardial coronary spasm in patients with angina and nonobstructive coronary arteries. The authors are to be congratulated for this well-conducted multicenter analysis addressing a crucial gap in our understanding of coronary vasomotor disorders.

The finding that both MB and moderate atherosclerosis are independent and additive predictors of epicardial spasm is clinically meaningful. The reported adjusted odds ratios (MB—odds ratio, 2.93; 95% CI, 1.67-5.13; moderate atherosclerosis—odds ratio, 2.32; 95% CI, 1.18-4.59) highlight the importance of recognizing structural contributors to vasospastic angina, even in patients without obstructive coronary disease.^1^

This study provides a timely opportunity to revisit the seminal work of Prinzmetal et al,^2^ who, in 1959, described variant angina as spontaneous chest pain at rest with transient ST-segment elevation due to reversible epicardial coronary spasm. Although Prinzmetal et al’s^2^ original description was based on clinical and electrocardiographic features, the work by Bastiany et al^1^ expands our understanding by integrating anatomical abnormalities with invasive physiological testing. This shift reflects the evolving concept that coronary spasm is not merely a functional disorder but often the result of complex interactions between structural and endothelial factors.

Mechanistically, the link between MB and spasm is supported by intracoronary imaging studies. Ge et al^3^ demonstrated that MB is associated with specific morphologic and hemodynamic features, such as the “half-moon sign” on intravascular ultrasound and the early diastolic “finger-tip phenomenon” on Doppler, both indicative of altered shear stress and flow dynamics. Moreover, Herrmann et al^4^ further established that MB sites exhibit impaired endothelium-dependent vasodilation and heightened wall shear stress, confirming a predisposition to vasomotor dysfunction.

Similarly, the association between atherosclerosis and vasospasm, as highlighted by Bastiany et al,^1^ is consistent with findings by Peeters et al,^5^ who demonstrated that patients with coronary spasm often exhibit a higher burden of nonobstructive atherosclerotic plaque, emphasizing the role of endothelial injury and inflammation as central mechanisms linking plaque and vasospasm.

We encourage future studies to incorporate systematic intracoronary imaging for improved MB characterization, use higher-dose acetylcholine protocols to assess multivessel spasm, and correlate angiographic findings with clinical phenotypes resembling Prinzmetal variant angina. Such efforts may further clarify the prognostic and therapeutic implications of these findings.

In conclusion, this study meaningfully advances the field by highlighting that structural coronary abnormalities and functional vasomotor disturbances frequently coexist and interact within the angina and nonobstructive coronary artery spectrum. The integration of anatomical and physiological assessments is essential for optimal patient care.
